# 

**Published:** 1853-08

**Authors:** 


VOL. II. NEW SERIES. No. 4.

### THE NORTH-WESTERN MEDICAL AND SURGICAL JOURNAL:

EDITED BY

W. B. HERRICK, M.D.,

PROFESSOR OF ANATOMY AND PHYSIOLOGY IN RUSH MEDICAL COLLEGE; SURGEON TO THE U. S. MARINE HOSPITAL, ONE OF THE SURGEONS OF THE ILLINOIS GENERAL HOSPITAL, ETC.

AND

H. A. JOHNSON, A.M., M.D.

PUBLISHED MONTHLY,

AT TWO DOLLARS PER VOLUME.

AUGUST, 1853.

CHICAGO:

BALLANTYNE & CO., PUBLISHERS & PRINTERS,

NEW POST-OFFICE BUILDINGS, COR. OF CLARK AND RANDOLPH-STS.,

OPPOSITE THE SHERMAN HOUSE.

1853.

VOL X. WHOLE SERIES. No. 4.

